# Characteristics associated with the risk of psychosis among immigrants and their descendants in France

**DOI:** 10.1002/brb3.2096

**Published:** 2021-04-09

**Authors:** Andrea Tortelli, Patrick Simon, Sophie Lehouelleur, Norbert Skurnik, Jean Romain Richard, Grégoire Baudin, Aziz Ferchiou, Marion Leboyer, Franck Schürhoff, Andrei Szöke

**Affiliations:** ^1^ INSERM U955 Translational Neuropsychiatry Créteil France; ^2^ Pôle GHU Psychiatrie Précarité Groupe Hospitalier Universitaire Paris Psychiatrie & Neurosciences Paris France; ^3^ Institut Convergences Migrations Aubervilliers France; ^4^ Institut National d’Etudes Démographiques Aubervilliers France; ^5^ Groupe Hospitalier Universitaire Paris Psychiatrie & Neurosciences Paris France; ^6^ Laboratoire de Psychopathologie et Processus de Santé Université de Paris EA 4057 Boulogne‐Billancourt France; ^7^ AP‐HP DMU IMPACT Hôpitaux Universitaires Henri‐Mondor Créteil France; ^8^ Fondation FondaMental Créteil France; ^9^ UPEC University Paris‐Est Créteil France

**Keywords:** epidemiology, migration, psychoses, public mental health, risk factors

## Abstract

**Objective:**

To explore the sociodemographic characteristics that might explain the increased incidence of psychosis among immigrants and their descendants in France.

**Methods:**

Data were collected for all subjects with first contact for psychosis aged between 18 and 64 years, in two catchment areas in the Paris region. Incidence rates (IR) and incidence rate ratios (IRR) were adjusted for gender and age.

**Results:**

During 805,396 persons‐year at risk, we identified 321 cases of first‐episode psychosis, of which 129 were immigrants and 78 descendants of immigrants. We found that the geographic origin was associated with the risk of psychosis although generation has little impact. Sub‐Saharan African immigrants and their descendants showed the highest risk (IRR = 3.1 and IRR = 2.9, respectively). We observed that living in deprived areas increased the incidence of psychosis (IRR = 1.3, 95CI%: 1.0–1.6), particularly among immigrants (IRR = 1.6; 95% CI: 1.1–2.5). Finally, our study showed that subjects having unstable housing (a proxy for “hard to count population”) could inflate the incidence rates among immigrants.

**Conclusion:**

The current study shows that the increased risk of psychosis in groups with an immigration background in France is associated with their origin and highlights the importance of socioeconomic factors in modulating this risk.

## Abstract 2

The aim of the present study is to explore the characteristics that might explain this increase: geographic origin, generation (immigrants or their descendants), social environment, and living conditions. We observed that living in deprived areas increased the incidence of psychosis, particularly among immigrants. Geographic origin was associated with the risk of psychosis although generation has little impact and Sub‐Saharan African immigrants and their descendants showed the highest risk.

## Significant outcomes

- Immigrants are at increased risk of psychosis in France and risk is most increased for Sub‐Saharan African immigrants and their descendants.
- Relative risk depends on the socioeconomic environment and is increased in a socioeconomical deprived area.
- “Hard to count populations,” that is, subjects with instable housing could artificially inflate incidence rates in migrants.

## Limitations

- Recruitment bias to barriers to access to mental health care for immigrants could not be ruled out.
- Reliability of data for immigrants descendants is impacted by the lack of data on ethnicity in the French Census.
- Due to limitations in the data available, we were unable to study incidence in descendants of internal migrants from French Overseas.

## 1 INTRODUCTION

Migrant and ethnic minority status have been associated with an increased risk of psychosis in many European countries (Bourque et al., 2011; Tortelli et al., 2015). A variation of this risk is observed as the result not only of premigration experiences (e.g., war, violence; (Hollander et al., 2016) but also as a result of experiences of social adversity in the host country such as discrimination, isolation, inequalities regarding welfare, health systems, employment, housing and legal status (Morgan et al., 2019; Selten et al., 2013).

France is one of the European countries with the longest history of immigration and the largest proportion of persons with an immigration background: in 2019, 6,7 million immigrants representing 9.9% of the population live in France; 46.5% were born in Africa (29.1% in North Africa and 17,4% in sub‐Saharan Africa); and 33.3% were born in Europe (mostly in Spain, Portugal, and Italy); and 11% of natives has at least one immigrant parent, that is, what is generally called a second‐generation immigrant. Refugees were estimated to be 300,000 in 2019 (UNHCR), 138,000 new applications were received that year, but only 26% of them were granted. Even though the recent migration flows are more diversified, the main origins remain similar in the last decades: 35% newcomers in 2017 came from Europe, 36% from Africa (North and sub‐Saharan Africa), and 18% from Asia. As in many countries, most immigrants live in urban areas, with 35% of first‐generation immigrants and 30% of second‐generation immigrants living in the Paris region (Ile de France; Brutel, 2017). In addition, internal migration from people born in the French Overseas departments *(*Guadeloupe et Martinique in the Caribbean, Guyana in South America, Mayotte and La Réunion in the Indian ocean) is also observed. They share a lot of characteristics with international immigrants, especially their skin color that make them vulnerable to discrimination and make up 1% of the population in mainland France, and two‐thirds of them live in the Paris region (Abdouni & Fabre, 2012).

Data on mental health disorders among migrant populations in France are scarce, and epidemiological studies in these populations have only recently been conducted. The new impetus of research on the subject is partly due to an increase in data availability on migrant populations in the last decade. The French census carried out by the National Institute of Statistics and Economic Studies (INSEE) as a rolling yearly estimate, collects data on country of birth but not on ethnicity as a variable itself. Public statistics (other than the census) can provide data on the descendants of immigrants but are still limited in number. Therefore, ethnic statistics are usually based on proxies such as country of birth and nationality of individuals and their parents, classification by first and/or last name, mother tongue or language spoken at home (Simon, 2010).

Furthermore, as in most countries, data on vulnerable migrants are even more difficult to collect. This is especially true when being an immigrant, or having a minority background, is coupled to a lack of legal status (Guyavarch & le Méner, 2014). Yet, the identification of these populations is particularly important given their increased vulnerability to mental disorders (Fazel et al., 2008).

In 2014, we published a first study on the association between migration and psychosis, analyzing first admissions for psychosis over 5 years in a 200,000‐people catchment area in Paris (20th district). Data indicated a higher admission rate among immigrants, as compared to natives (IRR = 2.9, 95% CI: 0.9–9.8), particularly among immigrants born in sub‐Saharan Africa (IRR = 7.1, CI: 95% 2.3–21.8) (Tortelli et al., 2014).

More recently, in a European multi‐national (six‐country) study of first contact for psychosis (EU‐GEI—European Gene‐Environment Interactions study; Van Os et al., 2014), we confirmed the association of increased risk of psychosis and migrant status in the same area in Paris (IRR = 2.21, 95% CI: 1.84–2.65) as well as in Val‐de‐Marne, a southern suburb of Paris (IRR = 1.99, 95% CI: 1.73–2.29; Jongsma et al., 2017). The treated incidence of psychosis among immigrants in France, when compared with the risk in the other participant countries, was among the highest. These data underscored the need for a better understanding of the differential risk of psychosis in France.

### 1.1 Aims of the study

Using data collected for in the EU‐GEI study, we decided to refine the analyses by investigating the incidence of psychosis by origin among immigrants and their descendants, and by considering the impact of the socioeconomic environment and living conditions on this risk. We hope that this study will represent a first step toward the design and implementation of tailored prevention policies and improved mental health care for the groups at risk.

## 2 MATERIALS AND METHODS

### 2.1 Ethical approval

The relevant Regional Ethical Committee (Comité de Protection des Personnes—CPP Ile de France IX) examined and approved the study protocol (project number 2010‐A00161‐38) in accordance with the Helsinki Declaration and its later amendments. The patients were not directly involved in the study and all the data sent to the research team preserved patient anonymity.

### 2.2 Setting

The study took place between 2012 and 2014 in the 20th district of Paris (199,790 inhabitants, 22% being immigrants) and between 2010 and 2014 in a metropolitan Paris suburb (204,304 inhabitants, 18% being immigrants), comprising the contiguous municipalities of Créteil, Boissy‐Saint‐Leger, Maisons‐Alfort, Sucy‐en‐Brie, and Bonneuil‐sur‐Marne.

### 2.3 Case ascertainment and data collection

Data were collected as part of the EU‐GEI study (Van Os et al., 2014). The sample was comprised of subjects aged between 18 and 64 years having a first contact for psychosis with a public psychiatric institution (in‐ or out‐patient) or with a private practitioner, living in the catchment areas for at least 6 months. In France, individuals with psychiatric disorders do not need to be referred by a general practitioner to have access to psychiatric facilities. In the case of psychosis, a referral is generally made to the public health system, especially in areas of medium or low socioeconomic status, as in the case of the catchment areas in our study. The diagnosis of psychosis was made according to DSM‐IV‐TR (American Psychiatric Association, 2000) and included nonaffective (codes 295.x, 297.x, 298.x) and affective psychotic disorders (codes 296.x4). Subjects with previous episodes of psychosis or with a probable organic cause of psychotic symptoms, including acute intoxication, were excluded.

A leakage study (based on the analysis of files of all first contacts with the in‐ and out‐patients psychiatric facilities of the catchment areas during the period of the study) to identify missing cases was carried out by a psychiatrist (AT) in both sites. All retrieved cases were reviewed by a senior psychiatrist/researcher who decided, based on available evidence, to include or to drop them from the dataset. Data were collected by age, gender, country of birth, and residential code by IRIS (Ilots Regroupés pour l'Information Statistique), a geographical area containing between 1,500 and 5,000 inhabitants).

### 2.4 Numerator

#### 2.4.1 Immigrants

Subjects born outside mainland France. The region of the country of birth was used as a proxy of ethnic origin, distributed in five groups representing the largest immigrant groups in France: Overseas France (mainly from the Caribbean “French West Indies,” Guadeloupe and Martinique), North Africa, Sub‐Saharan Africa, Europe (European Union), and Other (e.g., other European countries, Asia, the Americas).

#### 2.4.2 Descendants of immigrants

Subjects born in France to at least one immigrant parent. Since birthplace and nationality of the parents were not collected in this study, we had to use an onomastic method to estimate the ethnic origin of participants born in France. This procedure has been used as an alternative—or proxy, in the methodological terminology—when data for the ethnicity of the participant, or country of birth of their parents, are not available (Héran et al., 2010). Two researchers (PS, SL), who did not have access to the case notes, blind‐coded separate lists of the last names of participants born in France to a list of ethnic categories. To preserve the best fit, only four ethnic categories were defined: the majority group—France (including Overseas France, whose names are generally similar to participants from mainland France), North Africa, sub‐Saharan Africa, and Other (including Europe). Supplementary material (Internet, dictionary of names) was consulted by researchers when required to clarify last name origin. The two lists were then compared, with an agreement found for 186/188 cases (99%). A decision for the two remaining cases was made by agreement between the two researchers.

#### 2.4.3 Majority population

Subjects born in mainland France and of French origin, as defined by the onomastic method.

### 2.5 Denominator

#### 2.5.1 Immigrants

Data by migrant status were provided by the French census 2011 (place of birth). Migrant status and country of birth were available at municipality and IRIS levels. To match the numerator according to EU‐GEI inclusion criteria, age groups between 18 and 64 years were used.

#### 2.5.2 Descendants of immigrants

Since the parental place of birth is only identified in some surveys at a national level, estimates were provided by a national representative survey, TeO (Trajectories and origins; Beauchemin et al., 2016). This large‐scale survey was carried out in mainland France in 2008–2009 with almost 22,000 respondents (9,000 immigrants, 9,000 s‐generation immigrants, and 4,000 majority population) to measure the impact of origins on living conditions and social trajectories. The survey is representative of the different origin groups at the national and regional level, and for the Parisian region specifically at the departmental level. Proportions of immigrants and second‐generation immigrants were computed at the department level by age, gender, and ethnic background. The TeO data covered the age group from 18 to 50 years, and therefore we had to extend the coefficients of the oldest age group (45–50 years) to estimate the composition of the population for the age group 50 to 64 (Supporting Information 1).

#### 2.5.3 Majority population

Estimates were calculated by figures from the 2011 French census (individuals born in mainland France) from which we subtracted the number of subjects with foreign origin (data from TeO).

### 2.6 The socioeconomic environment

To investigate the impact of the social and economic environment on the incidence of psychosis, we studied IR and IRR in deprived areas called “priority neighbourhoods.” Priority neighborhoods are defined by the National Observatory of Urban Policy (INSEE, 2017) based on indicators of socioeconomic deprivation (proportions of low‐income households, unemployment, single‐parent families, study qualification, and social housing) and are located mostly in urban areas. In the 20th district of Paris and in the Val‐de‐Marne area analyzed in this study, around 40% of the total population live in such neighborhoods.

### 2.7 Data analysis

Data were separately analyzed for each site (Paris and Val‐de‐Marne) and by deprived /nondeprived areas.

Chi‐square tests were used to compare sociodemographic characteristics and diagnosis by migration background, unstable housing status, and ethnicity, for each site.

We calculated crude and age‐sex adjusted incidence rates (IR) and incidence rate ratios (IRR) with 95% confidence intervals (95% CI) and related probability (*p*), which are displayed relative to 100,000 person‐years in first‐ and second‐generation immigrants. To evaluate the impact of populations that are likely not counted in the census, we identified subjects with unstable housing status (hosted by acquaintances/relatives). IR and IRR were calculated with and without this group. Poisson regression was used to calculate adjusted IR and IRR by gender and age with 95% CI. Analyses were performed with Stata (version 14).

## 3 RESULTS

### 3.1 Sociodemographic characteristics

Three hundred cases were initially identified. The leakage study allowed the identification of additional 35 cases (7 cases in Paris and 28 cases in Val‐de‐Marne) and 6 cases were excluded because they showed to be false positives (not first contact for psychosis). Eight cases had missing data on country of birth. Therefore, the total cases analyzed are 120 in Paris and 201 in Val‐de‐Marne, among whom 28 were subjects with unstable housing status. The breakdown by migration background showed a total of 129 immigrants and 78 descendants of immigrants.

Of this sample, men were more represented at both sites. Immigrants were older (mean age = 35.0; *SD* = 12.1) than the majority group (mean age = 34.3; *SD* = 12.7) in both sites, and women were older (37.6 years; *SD* = 13.1) than men (29.8 years; *SD* = 10.2). The mean age of descendants of immigrants was 28.2 (*SD* = 9.8).

People with unstable housing status (26 in Paris and 2 in Val‐de‐Marne) were younger (mean age = 30.7; *SD* = 9.3), mostly men (82%) and immigrants (82%) from sub‐Saharan Africa (54%). If we exclude this group from the analyses, no statistically significant differences between sites related to the proportion of immigrants, minority ethnic group gender, or generation (*p* =.7) were found.

The “Other” group was constituted by subjects from Southeast Asia (Cambodia, Vietnam, Laos; *N* = 7), from Haiti (*N* = 5), from the Middle East (*N* = 6), from other European countries (*N* = 5), and the Americas (*N* = 2), China (*N* = 1), and Mauritius (*N* = 1). The “EU‐27” group (*N* = 6) was constituted by subjects from the United Kingdom, Poland, Portugal, and Romania (Tables 1 and 2).

**TABLE 1:** Demographic characteristics of cases included in the analyses

		Paris 20th				Val‐de‐Marne				Unstable housing status in the total sample	
		Total (%)	Majority population (%)	Immigrants (%)	Descendants of immigrants (%)	Total (%)	Majority population (%)	Immigrants (%)	Descendants of immigrants	Total (%)	Immigrant (%)
Total		120 (100)	36 (30)	59 (49.2)	25 (20.1)	201 (100)	78 (38.8)	70 (34.8)	53 (26.4)	28 (100)	24 (85.7)
Population at risk	273,656		115,710	89,143	68,803	531,740	240,150	168,543	123,047	—	—
Men		83 (69.2)	23 (63.9)	42 (83)	18 (72)	107 (53.2)	43 (55.1)	31 (44.2)	33(62.3)s	23 (82.1)	23 (82.1)
Age											
18–24		40 (33.3)	11 (30.5)	14 (23.7)	15 (60)	70 (34.8)	28 (35.9)	19 (27.1)	23 (43.4)	11 (39.9)	10 (41.6)
25–34		32 (26.7)	9 (26)	15 (25.4)	8 (32)	50 (24.9)	16 (20.5)	18 (25.7)	16 (30.2)	7 (25.0)	6 (25.0)
35–44		27 (22.5)	7 (19.4)	19 (32.2)	1 (4)	38 (18.9)	15 (19.2)	17 (24.3)	6 (11.3)	9 (32.1)	7 (29.1)
45–54		13 (10.8)	6 (16.7)	6 (10.2)	1 (4)	29 (14.4)	13 (16.7)	9 (12.8)	7 (13.2)	1 (3.5)	1 (4.1)
55–64		8 (6.7)	3 (8.3)	5 (8.5)	0 (0)	14 (6.9)	6 (7.7)	7 (10)	1 (1.9)	0 (0)	0ynn

**TABLE 2:** Immigrants: IR and IRR (95% CI) per 100,000 person‐years, adjusted for age and gender

Paris 20th and Val‐de‐Marne	Total sample					Unstable housing status excluded			
	Population at risk	N	Crude IR	Crude IRR (95% CI)	Adjusted IRR (95% CI)	N	Crude IR	Crude IRR (95% CI)	Adjusted IRR (95% CI)
Majority	355,860	114	32.0	—	—	110	30.1	—	—
Immigrants	257,686	129	50.0	1.6* (1.2–2.0)	1.7* (1.3–2.1)	105	40.1	1.3 (1.0–1.7)	1.4* (1.1–1.8)
Overseas France	26,397	11	41.7	1.3 (0.6–2.4)	1.3 (0.7–2.4)	11	41.6	1.3 (0.6–2.5)	1.3 (0.7–2.4)
North Africa	79,406	30	37.8	1.1 (0.7–1.8)	1.4 (0.9–2.1)	25	31.2	1.0 (0.6–1.6)	1.2 (0.7–1.8)
Sub‐Saharan Africa	57,092	55	96.3	3.0* (2.1–4.2)	3.1* (2.2–4.3)	42	73.6	2.4* (1.6–3.4)	2.4* (1.7–3.5)
EU−27	33,766	6	17.8	0.5 (0.2–1.2)	0.6 (0.2–1.4)	5	14.8	0.5 (0.1–1.1)	0.5 (0.2–1.3)
Other	61,022	27	44.2	1.4 (0.8–2.1)	1.5 (0.9–2.2)	22	36.0	1.2 (0.7–1.8)	1.2 (0.8–1.9)

Paris 20th	Total sample					Unstable housing status excluded			
	Population at risk	*N*	Crude IR	Crude IRR (95% CI)	Adjusted IRR* (95% CI)	*N*	Crude IR	Crude IRR (95% CI)	Adjusted IRR* (95% CI)
Majority	115,710	36	31.0	—	–	33	32.3	—	—
Immigrants	89,143	59	66.2	1.9* 1.3–2.8	2.4* 1.5–3.7	36	40.3	1.2 0.7–1.9	1.5 0.9–1–2.4
Overseas France	5,293	4	75.6	2.4 0.6–6.8	2.7* 1.0–7.7	4	75.5	2.6 0.7–7.4	2.8* 1.0–8.0
North Africa	29,531	13	44.2	1.4 0.7–2.7	1.7 0.9–3.4	8	27.1	0.9 0.4–2.1	1.1 0.5–2.3
Sub‐Saharan Africa	20,233	26	128.5	4.1* 2.4–7.0	4.4* 2.6–7.3	13	64.2	2.2* 1.1–4.4	2.3* 1.2–4.3
EU−27	11,929	4	33.5	1.1 0.2–3.0	1.2 0.4–3.4	3	25.1	0.8 0.2–2.8	0.9 0.3–3.1
Other	22,158	12	54.1	1.7 0.8–3.4	1.8 0.9–3.6	8	36.1	1.3 0.5–2.8	1.3 0.6–2.8

Val‐de‐Marne	Population at risk	*N*	Total sample			*N*	Unstable housing status excluded		
			Crude IR	Crude IRR (95% CI)	Adjusted IRR* (95% CI)		Crude IR	Crude IRR (95% CI)	Adjusted IRR* (95% CI)
Majority	240,150	78	38.3	—	—	77	38.0	—	—
Immigrants	168,542	70	41.5	1.1 0.8–1.5	1.3 0.9–1.2	69	40.1	1.1 0.8–1.4	1.3 0.9–1.9
Overseas France	21,104	7	33.1	1.0 0.4–2.2	0.9 0.4–2.1	7	33.1	1.0 0.4–2.2	0.9 0.4–210
North Africa	49,875	17	34.1	1.0 0.6–1.8	1.2 0.7–2.1	17	34.1	1.1 0.6–1.8	1.2 0.7–2.1
Sub‐Saharan Africa	36,860	29	78.7	2.4* 1.5–3.7	2.4* 1.6–3.7	29	78.7	2.4* 1.5–3.8	2.5* 1.6–3.8
EU‐27	21,837	2	9.0	0.3* 0.1–1.0	0.3 0.1–1.3	2	9.1	0.3* 0.1–1.0	0.3 0.1–1.4
Other	38,864	15	38.6	1.2 0.6–2.1	1.2 0.7–2.1	14	36.0	1.1 0.6–2.0	1.1 0.6–2.1

Note

Paris versus Val‐de‐Marne: origin (*p* =.81).

* *p* <.05.

### 3.2 Incidence of psychosis

In the total sample (Paris and Val‐de‐Marne), the incidence was 39.8/100,000 person‐years (population at risk = 805,396). The incidence among the majority group was 32.0/100,000 person‐years. The immigrant group showed an IR of 50/100,000 person‐years and among their descendants, the IR was 40.6/100,000 person‐years. When compared to the majority population we found an adjusted IRR = 1.7 (95% CI: 1.3–2.1) among immigrants and an IRR = 1.1 (95%CI: 0.8–1.5) among their descendants (Tables 2 and 3).

**TABLE 3:** Descendants of immigrants: IR and IRR (95% CI) per 100,000 person‐years, adjusted for age and gender

Paris 20^th^						Val‐de‐Marne					Total Sample		
	Person‐years at risk	Cases* (*N*)	Crude IR	Crude IRR (95% CI)	Adjusted IRR* (95% CI)	Person‐years at risk	Cases (*N*)	Crude IR	Crude IRR (95% CI)	Adjusted IRR* (95% CI)	Crude IR	Crude IRR (95% CI)	Adjusted IRR* (95% CI)
Majority	115,710	33	28.5	—	—	240,150	77	32.06	—	—	30.9	—	—
Descendants of immigrants	68,803	25	36.3	1.2 (0.7–2.2)	1.0 (0.6–1.7)	123,047	53	43.0	1.3 (0.9–1.9)	1.2 (0.8–1.7)	40.6	1.3 (0.9–1.7)	1.1 (0.8–1.5)
North Africa	23,526	13	55.2	1.9 (0.9–3.7)	1.7 (0.9–3.3)	41,983	17	40.4	1.2 (0.7–2.1)	1.1 (0.7–2.0)	45.8	1.4 (0.9–2.2)	1.3 (0.9–2.0)
Sub‐Saharan Africa	5,125	10	195.1	6.8* (3.0–14.2)	3.8* (1.8–8.0)	10,543	12	113.8	3.5* (1.7–6.5)	2.4* (1.2–4.4)	140.4	4.5* (2.7–7.2)	2.9* (1.8–4.6)
Other*	40,152	2	4.9	0.1 (0.0–0.6)	0.1 (0.0–0.6)	70,521	24	34.0	1.0 (0.6–1.7)	0.9 (0.6–1.5)	23.4	0.7 (0.0–1.1)	0.6 (0.4–1.0)

Note

Subjects with unstable housing status excluded.

* *p* <.05.

The incidence among immigrants was higher in the Paris site (66.2/100,000 person‐years) than in Val‐de‐Marne (41.5/100,000 person‐years). The adjusted IRR between immigrants and the majority group amounted to 2.4 (95% CI: 1.5–3.7) in Paris and 1.3 (95% CI: 0.9–1.2) in Val‐de‐Marne. If we excluded people with unstable housing status, the IRR in Paris decreased considerably to 1.5 (95% CI: 0.9–2.4). By place of birth, sub‐Saharan Africans showed the highest IRR of psychosis in both sites (Paris: IRR = 4.4; 95% CI: 2.6–7.3, and Val‐de‐Marne: IRR = 2.4; 95% CI: 1.6–3.7) followed by subjects from Overseas France living in Paris (IRR = 2.7; 95% CI: 1.0–7.7; Table 2).

Among French‐born participants (*N* = 192), seventy‐eight were classified as from immigrant descent, through the onomastic method (Table 3). Persons born in Overseas France were not specifically identified and thus were included in the majority population group (participants born in France with French‐origin last names). Sub‐Saharan African descendants comprised the only group showing a statistically significant higher incidence of psychosis than the majority group in both nonadjusted (IRR = 4.5; 95% CI: 2.7–7.2) and adjusted (IRR = 2.9; 95% CI = 1.8–4.6) models.

### 3.3 The impact of the social environment on the incidence of psychosis

Analyses in this section were conducted without the people with unstable housing status and subjects (*N* = 2) which neighborhoods could not be classified as deprived or not (Table 4). Sociodemographic characteristics of neighborhoods at both sites are shown in Supporting Information 2.

**TABLE 4:** Risk of psychosis in deprived areas (Paris and Val‐de ‐ Marne): IR and IRR per 100,000 person‐years, adjusted for age and gender

	Deprived areas				Nondeprived areas				Between areas
Migrant status	Cases (*N*)	Crude IR	Crude IRR (95% CI)	Adjusted IRR (95% CI)	Cases *N*	Crude IR	Crude IRR (95% CI)	Adjusted IRR* (95% CI)	Adjusted IRR
Total	139	47.6	—	—	152	35.8	—	—	1.3* (1.0–1.6)
Mainland France	74	42.3	—	—	113	36.8	—	—	1.1 (0.8–1.4)
Immigrants	65	55.6	1.3 (0.9–1.8)	1.7* (1.2–2.4)	39	33.1	0.8 (0.6–1.3)	1.1 (0.7–1.6)	1.6* (1.1 2.5)
Overseas France	10	77.1	1.8 (0.8–3.5)	2.3 * (1.1–4.5)	1	8.4	0.2 (0.0–1.3)	0.2 (0.0–1.7)	9.6* (1.2–76.2)
Europe	2	18.7	0.4 (0.0–1.6)	0.6 (0.1–2.5)	3	14.7	0.3 (0.0–1.1)	0.5 (0.1–1.6)	1.3 (0.2–7.8)
North Africa	16	42.1	0.9 (0.5–1.7)	1.3 (0.7–2.4)	9	25.2	0.6 (0.3–1.3)	0.9 (0.4–1.8)	1.6 (0.7–3.6)
Sub‐Saharan Africa	27	95.6	2.2 (1.3–3.5)	2.8* (1.8–4.4)	15	72.9	1.9 (1.0–3.4)	2.2* (1.3–3.9)	1.3 (0.7–2.5)
Other	10	37.1	0.8 (0.4–1.7)	1.1 (0.5–2.1)	11	37.4	1.0 (0.4–1.8)	1.2 (0.6–2.2)	0.9 (0.4–2.3)

Note

Subjects with unstable housing status excluded.

* *p* <.05.

The incidence of psychosis was higher in deprived areas (47.6/100,000) than in nondeprived areas (35.8/100,000; IRR = 1.3, 95% CI: 1.0–1.6). Among immigrants, this ratio was 1.6 (95% CI: 1.1–2.5). Of note, the risk among immigrants was no longer statistically significant in nondeprived areas (IR = 1.1; 95% CI: 0.7–1.6). By place of birth, the risk of psychosis among sub‐Saharan immigrants remained higher compared with that of subjects born in mainland France in both deprived (IRR = 2.8; 95% CI: 1.8–4.4) and nondeprived areas (IRR = 2.2; 95% CI: 1.3–3.9) and not statically different between areas (IRR = 1.3; 95% CI: 0.7–2.5). Notwithstanding the small sample sizes, subjects from Overseas France, who lived mostly in deprived areas, showed an increased risk (IRR = 2.3; 95% CI: 1.1–4.5) in comparison with the reference group (mainland France).

## 4 DISCUSSION

### 4.1 Principal findings

Using data collected in France for the EU‐GEI study, in this paper, we aimed to estimate the risk of psychosis by origin among immigrants and their descendants compared with the majority population. We also explored the impact of the social environment and living conditions on the variations of the incidence of psychosis.

We found that Sub‐Saharan African immigrants and their descendants are at increased risk of psychosis. Immigrants from the French Overseas also show a higher risk of psychosis in different contexts (in Paris and deprived areas), but we were unable to assess this risk among their descendants.

When we considered the socioeconomic environment, we observed that the association between increased risk of psychosis and immigrant status was present only in deprived areas, except for immigrants from sub‐Saharan Africa, who were at increased risk in both deprived and nondeprived areas. Importantly, the identification of subjects with unstable housing allowed to estimate the impact of a proxy of “hard to count population” in the incidence of psychosis in our sample.

### 4.2 Comparison with previous studies

Our findings are in accordance with our previous study on first admissions for psychosis conducted in Paris (Tortelli et al., 2014) showing an increased risk of admission for psychosis among sub‐Saharan Africans, but not for North Africans (as it is observed in the Netherlands, Velinget al., 2006). Our findings on French Overseas immigrants (notwithstanding methodological issues discussed above) are also in line with other European studies showing an increased risk among immigrants from the Caribbean (Tortelli et al., 2015), and with an increased prevalence of psychosis in the French Overseas and Mainland France (Amad et al., 2013; Ballon et al., 2004).

The increased incidence of psychosis in deprived areas is consistent with a large literature showing an association between social adversity and psychosis (Allardyce & Boydell, 2006; Bhavsar et al., 2014; Kirkbride et al., 2012). In our sample, the number of subjects with unstable housing status (8.7%) was higher than found in the general population in Ile de France (<1%; Pierre‐Marie et al., 2014) and mostly from sub‐Saharan Africa, which is accordance with a study carried out among people living in social accommodations in Paris showing that this group constituted the majority of the residents (Vandentorren et al., 2016).

### 4.3 Strengths and limitations

A leakage study allowed the identification of missing cases, thereby improving data quality. Although the numerator data used in the present study are derived from the same database as that of the multi‐national study comparing the risk among immigrants across different countries (Jongsma et al., 2017), we followed a different method to estimate the risk of psychosis in our study. In the multi‐national study, the UK population was used as a reference to adjust for age and gender. In our study, we could use local denominator data and we included Overseas people in the immigrant group. We brought the analysis a step further by calculating the risk among descendants and analyzed data regarding social conditions and environment, which improved the understanding of the results.

We cannot exclude that findings were influenced by methodological issues (Dorling & Thomas, 2004). First, a potential recruitment bias cannot be ruled out; although the study attempted to contact all private practitioners and institutions, the possibility of missing cases cannot be excluded, particularly in Paris. This bias might have increased the proportion of participants with low socioeconomic status, especially immigrants with unstable housing status. Barriers about access to mental health care for migrant populations might have influenced case ascertainment in migrant populations as well older age at first contact (Cooper et al., 2013).

Secondly, the methods used in our study to estimate the denominator and the numerator of descendants of immigrants are less reliable than for immigrants. Although the coefficients applied to the population of the catchment areas are based on a representative national sample, their relative lack of precision might entail differences in the proportion of first‐ and second‐generation ethnic groups overall and in the breakdown by gender and age. Further, for this analysis, we had to include the Overseas France group in the French majority group. The onomastic method has limitations as a proxy of ethnicity. The analysis of the last name seems to be more reliable due to mobility and “invisibility” strategies used in the choice of first names (Toar et al., 2009). Moreover, we could not rule out that some of the “second‐generation” subjects were in fact, third‐ (or more) generation. However, non‐European third‐generation immigrants are still quite young and mostly below 15 years old. Additional bias leading to misestimating results among descendants of immigrants may also be expected due to name shifting, mainly as a consequence of intermarriage. As a reference, 13% of all unions involve French‐born and migrant partners, with approximately 50% of migrant marriages made from within the same ethnic group (Collet & Régnard, 2008). Finally, the over‐representation of Sub‐Saharan Africans among subjects with unstable housing status suggests underestimation of the denominator for this group, as a hard‐to‐count population, which partly accounted for the increased risk among immigrants in Paris in comparison with the Val‐de Marne.

The impact of reverse causality on the increased psychosis risk in deprived areas cannot be excluded either, driven by people with psychosis showing social drift, that is, downward residential mobility to lower socioeconomic neighborhoods. Yet, in the Parisian region, the time frame for a single adult to access social housing (50% of housing choices in these neighborhoods) is about 10 years, which makes the social drift hypothesis less probable in first‐episode psychosis.

Another limitation of the current study is that it does not directly test issues of possible misdiagnosis in migrant populations in the French context. Misdiagnosis has been an ongoing concern, as the result of the use of a Western disease model (Fung et al., 2009), although this may be less relevant with the use of operationalized and standardized diagnostic criteria that have been validated across countries and cultures (Selten & Hoek, 2008). Moreover in France, especially in Ile the France, there is a considerable number of psychiatrists of African origin working in psychiatric wards which may have decreased this risk, similar to what has been observed by Hickling and colleagues in South London (Hickling et al., 1999). Yet, misdiagnosis may also be the result of cultural stigma against ethnic minority groups, through the over/misinterpretation of their behavior and beliefs (Fernando, 1991). On the other hand, some studies showed differences in the prevalence of positive and negative symptoms such as visual hallucinations (Suhail & Cochrane, 2002) or catatonia (Jablensky et al., 1992) between occidental and nonoccidental cultures.

The lack of data on ethnicity, religious background, and housing conditions in the French statistics did not allow to discard the impact of these factors on the incidence of psychosis. Also, the identification of homeless and people living in unstable housing in the census is uneven. For this reason, it was not possible to compute specific IRR for this sub‐population and we decided not to include them in the analysis.

### 4.4 Interpretation of findings

Inequalities in health, social trajectories, and access to resources between immigrants and their descendants and the native population have been recently highlighted and detailed in a large national survey (TeO—Trajectories and Origins; Beauchemin et al., 2016). The increased risk of psychosis among sub‐Saharan Africans in our study is supported by data showing that this group is more likely to be exposed to experiences of discrimination in daily life and access to higher education, employment opportunities, and housing, in comparisons with other immigrants and their descendants (including internal immigrants from Overseas France; Brimbaum et al., 2016; Safi & Simon, 2014). Sub‐Saharan Africans also account for most of the homeless people (including families) living in the Paris area, estimated about 40,000 in 2012 (Guyavarch & le Méner, 2014; Yaouancq & Duée, 2014), which is consistent with our findings of subjects without stable housing. French political (immigration and integration policies) contexts may also have contributed to the different social outcomes of immigrants (Brimbaum et al., 2016; Grosfoguel, 1997; Simon, 2015). For instance, factors favoring socioeconomic integration in times of economic prosperity in the 1970s have lost their potential with economic restructuring, while immigration law restrictions throughout the 1980s to the 2000s have increased the number of irregular statuses and homelessness among migrants (le Courant, 2015; le Méner & Oppenchaim, 2012). Unauthorized immigrants are estimated (regarding access to the state medical insurance) to be about 300,000, generally concentrated in urban centers, again represented by sub‐Saharan Africans in 50% of cases (Boisguérin & Haury, 2008).

Moreover, studies show that the “healthy‐migrant effect,” that is, an initial better health status than natives, tends to dissipate over the years spent in France, leading to lower levels of general health in immigrants (Berchet & Jusot, 2010; Hamel & Moisy, 2016). For instance, a recent study also found an excess in mortality for second‐generation North African, specifically male, that remains significant after adjusting for age and education (Anderson et al., 2015).

Taken together, our findings support the impact of cumulative psychosocial factors in the host country on the incidence of psychosis for a given ethnic group in each context, rather than the migrant status per se (Morgan et al., 2019; Stilo & Murray, 2010). Cumulative bio‐psychosocial stress has been suggested to be associated with the sensitization of the dopamine system, and then to contribute to the development of psychiatric disorders (Mothersill & Donohoe, 2016), including psychosis (Howes et al., 2017). In this line, the higher risk of psychosis among darker skin ethnic groups (Sub‐Saharan and French Overseas) is congruent with the international literature (Qassem et al., 2015), suggesting additional exposure to social risk factors such as discrimination and racism (Brimbaum et al., 2016; Sellers et al., 2003; Veling et al., 2007), and possibly environment‐driven biological changes, such as vitamin D deficiency (Anderson & Maes, 2013; McGrath et al., 2010).

Also, cultural distance, as a cause of psychological stress, has been put forward as one explanation of the increased risk of psychiatric disorders (Devylder et al., 2013; Hwang et al., 2008). For instance, in the EU‐GEI study, the linguistic distance was associated with an increased risk of psychosis (Jongsma et al., 2020). In the case of the French context, this seems less likely, since the main minority ethnic groups (North African, sub‐Saharan African, and French Overseas) come from former French colonies and at the same time, show different risks. Religious background/differences have also been shown to be related to psychological distress (Jordanova et al., 2015), particularly among Muslims, and unrelated to religion attachment level (Joly & Reitz, 2018). Yet, in our study, North Africans are not at increased risk of psychosis. Moreover, the positive link between psychological distress and cultural distance is also influenced by the perception of the majority group of multiculturalism as threatening and personal support toward integration models (Mahfud et al., 2016).

Finally, the role of selective migration (the hypothesis which holds that people predisposed to psychosis migrate as a consequence of poor social adjustment and integration in the country of birth) (Ødegaard, 1932) has been excluded as the main hypothesis of the increased risk of psychosis in migrants (Selten et al., 2002; van der Ven et al., 2015). However, it could not be discarded as a possible explanation of the increased risk among subjects from the French Overseas similar to what was observed by Tarricone and colleagues among internal migrants in Italy (Tarricone et al., 2016). Indeed, internal migration from Overseas departments may not involve the same challenges (e.g., high levels of planning skills and management of emotions, administrative barriers, acculturation aspects) than the migration from Africa and is usually facilitated by acquaintances and family members who live in mainland France. Given the small sample size, before any definitive conclusion could be drawn, this finding needs to be replicated.

Still, further investigation is required to continue to identify environmental and individual factors and their interactions that differentially influence the incidence of psychosis in immigrants and their descendants. For instance, premigration factors are rarely investigated in studies among migrants, except those focusing on refugees and asylum seekers. At the same time, there is also the need to better understand the protective factors underlying the ethnic density effect, such as social capital and cohesion. Finally, there is a lack of data on biological factors potentially associated with the development of psychosis among immigrants.

Hopefully, a better understanding of the causes—social, economic, or biologic—of the excess of psychosis in migrant groups will impact interventions to prevent psychosis but also the stigma that might be attached to a higher vulnerability to severe mental disorders.

### 4.5 Conclusion

We found an increased risk of psychosis among migrants and their descendants. Sub‐Saharan Africans were significantly more at risk than the other minority ethnic groups. This study highlights the importance of including the social context and consider the “hard‐to‐count” populations, to assess the risk of mental disorders and suggest potential causes more accurately. As such, our study might be a first step in understanding the origin of the excess of psychotic disorders in the migrant populations in France. Follow‐up and more specific studies are needed to tease out specific factors/potential causes that might explain the higher rates of psychosis in migrants such as potential misdiagnosis, selective migration, economic hardship (e.g., unstable housing), or social adversity (discrimination).

## CONFLICT OF INTEREST

None.

## **AUTHOR** **CONTRIBUTION**

AT, PS, AS, and FS: Conceptualization and study design. AT, GB, and AF: Data acquisition. JRR: Data curation. SL and AT: Data Analysis. AT and PS: Writing the first draft. AT, PS, AS, FS, NS, and ML: Reviewing and editing the manuscript. AS, FS, and ML: Funding acquisition. All authors read and approved the final manuscript.

### Peer Review

The peer review history for this article is available at https://publons.com/publon/10.1002/brb3.2096.

## Supporting information

Supplementary Material

## Data Availability

The data that support the findings of this study are available from the corresponding author upon reasonable request.
