# Supplementary material for: Characteristics associated with the risk of psychosis among immigrants and their descendants in France
Source: Brain Behav. 2021 Apr 9;11(5):e02096. doi: 10.1002/brb3.2096 (PMC8119809; doi:10.1002/brb3.2096)
Supplement: Supplementary file 1 — Supplementary Material [file BRB3-11-e02096-s001.docx]

**Supplementary material 1**

**Coefficients used for second generation denominator estimation**

| **Gender** | **Age class** | **North Africa** | **Sub-Saharan Africa** | **Other** |
| --- | --- | --- | --- | --- |
| **Women** | 17-24 | 2.970729291 | 0.954318811 | 2.8514238 |
|  | 25-34 | 1.484432229 | 0.410597345 | 1.139887125 |
|  | 35-44 | 0.898656149 | 0.096826886 | 0.843176196 |
|  | 45-50 | 0.434339903 | 0.048735067 | 0.740367146 |
| **Men** | 17-24 | 3.240858614 | 1.847339555 | 3.497823574 |
|  | 25-34 | 1.422711557 | 0.396268616 | 1.504586084 |
|  | 35-44 | 0.744484597 | 0.182037451 | 1.056742856 |
|  | 45-50 | 0.364138997 | 0.062943088 | 1.014005159 |

***Source :* TeO and INSEE 2011.**

**Supplementary material 2**

|  | **Paris 20^th^** | | **Val-de-Marne** | |
| --- | --- | --- | --- | --- |
|  | **Deprived areas**  % | **Non- deprived areas**  % | **Deprived**  **areas**  % | **Non- deprived areas**  % |
| **Immigrants** | 26.6 | 19.0 | 23.7 | 17.5 |
| **Single - parent families** | 28.2 | 19.9 | 27.9 | 17.2 |
| **Minimum social income** | 21.5 | 17.6 | 19.0 | 12.9 |
| **Unemployment** | 17.0 | 14.1 | 17.8 | 10.9 |
| **No study qualification** | 23.0 | 13.5 | 25.1 | 16.4 |
| **15-17y with no secondary school level** | 3.4 | 1.3 | 4.5 | 2.5 |
| **Social housing** | 53.4 | 24.3 | 67.8 | 24.0 |
|  |  |  |  |  |

**Sociodemographic characteristics of deprived and non-deprived areas**

***Source:*  National Observatory of Urban Policy (INSEE 2017)**
